# Pathology, Diagnosis, and Treatment of Diseases of Women

**Published:** 1888-07

**Authors:** 


### BOOK NOTICES AND REVIEWS.

THE PATHOLOGY, DIAGNOSIS, AND TREATMENT OF THE DISEASES OF WOMEN. By Graily Hewitt, M. D., F. R. C. P. Edited, with notes, by H. Marion Sims, M. D. Three volumes; $2.75 per volume. E. B. Treat & Co., New York, 1887.

Professor Hewitt has long been known as one of the leading teachers of gynæcology in England. His teaching has been of a conservative nature, relying rather upon dietetics, the rectifying of mal-positions of uterus, and internal medication than upon the more heroic surgical methods of his confreres. As a broad axiom, he states that the *local* diseases are dependent upon *general* causes. Most of the hysterical and other diseases of woman he attributes to uterine displacement, etc., rather than to ovarian disease. This may be true, yet it must be confessed that the ovaries exercise a very great influence over the nervous life of the women.

Dr. Hewitt's work is well illustrated, and is perhaps one of the few books of this class that the general practitioner should carefully study. In his ideas upon the pathology of these diseases, Dr. Hewitt is more in harmony with the views of homœopathic pathology than any others of his school.

